# 

**Published:** 1858-07

**Authors:** 


### THE SANITARY REVIEW.

### CONTENTS OF No. XIV.

Hospital Hygiene . . . . . . . . 113
The Social Science Transactions . . . . . . 129

**EPITOME OF SANITARY LITERATURE:**

Lee (Edwin). Climate . . . . . . . 137

Gant (F. J.) Diseased Prize Cattle . . . . . *ib*

Pinkerton (A. P. W., M.D.) Cholera . . . . 138

Lankester (E., M.D., F.R.S.) Drinking Waters of London . . *ib.*

Guy (W. A., M.B.) Mortality of the Army . . . . 139

Howe (S. G., M.D.) Causes of Idiotcy . . . . 140

Pickford (J. H., M.D.) Hygiene . . . . 141

Miscellaneous Works . . . . . . *ib.*

**SANITARY AND SOCIAL SCIENCE.**

The Thames . . . . . . . 142
Ventilation of St. James's Hall, Piccadilly . . . . 144

**ORIGINAL COMMUNICATIONS:**

Notes on the Topography, Meteorology, and Sanitary Condition of Sinope. By J. N. Radcliffe, Esq. . . . . . . 145
Child-Murder in its Sanitary and Social Bearings. By W. B. Ryan, M.D.Lond. . . . . . . 165
Remarks on the Importance of avoiding the Contamination of Water with Lead. By J. B. Harrison, M.D. . . . . 185

**PROGRESS OF EPIDEMICS** in Forty-one Towns and Districts, contributed specially, by the Staff of Local Observers . . . . 187

**SANITARY LEGISLATION** . . . . . . 207

**TRANSACTIONS of the EPIDEMIOLOGICAL SOCIETY of LONDON:**

On the Sickness and Mortality in the French Army, during the Campaign in Turkey and the Crimea in 1854-56 . . . . . 21
Annual Report of the Council . . . . . 44

